# Reviewed article: Mour EB, Catelano BA, Aguiar FP, Lima H, França PHC. Survival of critically ill people with COVID-19 and acute kidney injury undergoing hemodialysis in public and private hospitals in Joinville: a cohort study, 2020-2021. Epidemiol Serv Saude. 2025:34;20240025. doi: 10.1590/S2237-96222025v34e20240025.en

**DOI:** 10.1590/S2237-96222025v34e20240025.b

**Published:** 2025-04-07

**Authors:** Thaynã Ramos Flores

**Affiliations:** 1 Universidade Federal de Pelotas, Pelotas, RS, Brazil.

## Peer review B

## Questionnaire

Does the manuscript contain new and significant information to justify publication? Yes

Does the Abstract (Summary) clearly and accurately describe the content of the article? No

Is the problem significant and concisely stated? No

Are the methods described comprehensively? No

Are the interpretations and conclusions justified by the results? No

Is adequate reference made to other work in the field? Yes

## Manuscript Structure

Length of article is: Adequate

Number of tables is: Adequate

Number of figures is: Adequate

**Please state any conflict(s) of interest that you have in relation to the review of this paper (state “none” if this is not applicable).**

None

**Interest:** Good

**Quality:** Average

**Originality:** Average

**Overall:** Average

**Recommendation:** Major Revision

## Comments

Trata-se de um estudo cujo objetivo foi comparar a sobrevida (em 90 dias) de pacientes críticos com COVID-19 e IRA que estavam em UTI de hospitais públicos e privados. A pesquisa pode contribuir para o melhormanejo e destinar uma atenção redobrada para pacientes com essas condições, mesmo que a pandemia deCOVID-19 tenha sido satisfatoriamente controlada pela vacinação. O manuscrito aborda uma temática interessante, porém possui lacunas que devem ser resolvidas para queseja considerado para publicação na RESS.

Abaixo listo minhas principais preocupações que podem contribuir para o aprimoramento do estudo:

1. o delineamento do estudo necessita de revisão e adequação; não fica claro porque os autoresconsideraram uma “coorte histórica”;
2. a justificativa do estudo tem condições de ser aprimorada para que mencione claramente quais asprincipais contribuições deste estudo e o que ele pode acrescentar de novidade para literatura sobre essatemática;
3. na introdução há evidências da avaliação da sobrevida em 60 dias, mas os autores não explicam aescolha dos 90 dias para avaliação neste estudo;
4. a amostragem do estudo não foi adequadamente descrita na seção de métodos; essa seção (métodos)deve ser abordada com maior detalhamento permitindo a replicação do estudo por outros pesquisadores daárea; revisar a recomendação STROBE (http://www.scielo.br/pdf/rsp/v44n3/21.pdf);
5. há necessidade de mencionar qual(is) a(s) variável(is) de desfecho de interesse do estudo, nos métodos;
6. é importante mencionar quais variáveis foram utilizadas no modelo multivariado;
7. as limitações foram descritas, mas os autores não discutem elas mencionando como lidaram compossíveis vieses ocasionados por esses problemas;
8. o processo de consentimento informado não foi descrito com detalhamento. Necessário incluir essasinformações também.

Comentários gerais:

• O manuscrito necessita de ajustes com relação ao atendimento às normas da RESS. As características dacidade do estudo não foram mencionadas nos métodos, por exemplo. O objetivo ao final da introdução não éo mesmo do resumo, entre outros aspectos que podem ser destacados aos autores considerando as normasda revista. Além disso, acredito que a pergunta de pesquisa deveria ser mais bem definida.
• As conclusões do estudo, sobretudo do resumo, devem ser revisadas para garantir que sejam justificadaspelos achados e respondam diretamente à pergunta de pesquisa.
• Ainda, sugiro que os autores expliquem como os resultados podem ser generalizados explicando a validadeexterna do estudo.
• O texto necessita de ampla revisão, utilizando linguagem clara, concisa e formal (evitar conjugação verbalna primeira pessoa).
• As ilustrações também necessitam de revisão, especialmente quanto às normas e diretrizes da RESS.

